# Upscaled CTAB-Based DNA Extraction and Real-Time PCR Assays for *Fusarium culmorum* and *F. graminearum* DNA in Plant Material with Reduced Sampling Error

**DOI:** 10.3390/ijms9112306

**Published:** 2008-11-25

**Authors:** Christoph Brandfass, Petr Karlovsky

**Affiliations:** University of Göttingen, Department of Crop Sciences, Molecular Phytopathology and Mycotoxin Research Division, Grisebachstrasse 6, 37077 Göttingen, Germany

**Keywords:** *Fusarium graminearum* Schwabe, *Fusarium culmorum* W.G. Smith, real-time PCR, DNA extraction, sampling error, Fusarium head blight

## Abstract

*Fusarium graminearum* Schwabe (*Gibberella zeae* Schwein. Petch.) and *F. culmorum* W.G. Smith are major mycotoxin producers in small-grain cereals afflicted with Fusarium head blight (FHB). Real-time PCR (qPCR) is the method of choice for species-specific, quantitative estimation of fungal biomass in plant tissue. We demonstrated that increasing the amount of plant material used for DNA extraction to 0.5–1.0 g considerably reduced sampling error and improved the reproducibility of DNA yield. The costs of DNA extraction at different scales and with different methods (commercial kits versus cetyltrimethylammonium bromide-based protocol) and qPCR systems (doubly labeled hybridization probes versus SYBR Green) were compared. A cost-effective protocol for the quantification of *F. graminearum* and *F. culmorum* DNA in wheat grain and maize stalk debris based on DNA extraction from 0.5–1.0 g material and real-time PCR with SYBR Green fluorescence detection was developed.

## 1. Introduction

Fusarium head blight (FHB) is a disease of cereal crops with a severe impact on wheat and barley production worldwide. The infection of heads of small grain cereals and maize plants with *Fusarium* spp. impairs both grain yield and quality [1–2]. Apart from adversely effecting the grain size, weight, protein content, baking quality of the flour and other technological parameters, a serious consequence of FHB is the contamination of grain and cereal products with *Fusarium* mycotoxins [3]. Because grains of low quality are used in feedstuff production rather than for human foods, health damage in farm animals and losses in meat production caused by mycotoxin contamination of feeds have regularly been reported [4]. Low doses of mycotoxins may pose a risk to human health when consumed over prolonged time periods [5]. Two trichothecene mycotoxins produced by *Fusarium* spp. are regarded as bioterrorist threat agents [6]. Many countries have established legal limits for *Fusarium* mycotoxins in food and feeds.

In spite of breeding for FHB-resistance [7–9] and prioritization of fungicide development towards FHB, the disease continues to pose a major challenge to grain growers all over the world. A key prerequisite for FHB reduction through crop management is to understand the etiology of the disease and the biology of its causative agents. *Fusarium* species primarily involved in FHB are *F. graminearum* Schwabe and *F. culmorum* W.G. Smith [10]. The biology and the infection mode of these two species differ: *F. graminearum* reaches cereal heads via ascospores of the teleomorph *Gibberella zeae* forcibly discharged from asci formed on plant residues on the soil surface, while *F. culmorum* does not possess a sexual cycle. *F. culmorum* presumably reaches the ears by traversing from one leave to the next in rain splashes [11], but some researchers find this hypothesis unsatisfactory [12]. A hypothesis that *F. culmorum* and possibly also *F. graminearum* can infect cereal plants systemically and grow within the stem from the root up to the ear has been revived in recent years [13]. Another important question in FHB etiology is whether species other than *F. graminearum* and *F. culmorum* contribute significantly to the symptoms of FHB and mycotoxin contamination. A number of fungal species have been isolated from infected or even healthy-looking ears collected in the field, including a plethora of *Fusarium* spp. [2, 14], but it is not known whether all these species actively contribute to the FHB or whether they just grow saprophytically in dead tissues on the surface of the ear.

Deoxynivalenol (DON) and nivalenol (NIV) are the major representatives of trichothecene mycotoxins in grain. The level of trichothecenes and the amount of *F. culmorum* and *F. graminearum* DNA correlate well, indicating that these two species are largely responsible for the presence of the trichothecenes. The correlation coefficient for DON and DNA content was reported to be 0.68 [14] and 0.91 [15] for the sum of *F. culmorum* and *F. graminearum* DNA, and 0.96 or 0.75 (depending on the concentration range) for *Fusarium* species containing biosynthetic genes for DON [16]. Similarly, NIV content and the amount of *F. poae* DNA correlated tightly in samples from Finland and Russia [17].

The relative contribution of each *Fusarium* species to the disease in a particular situation depends upon a range of variables, which might also affect the amount of mycotoxins produced. Investigation of the etiology of fungal disease often requires quantitative determination of the biomass of the pathogen within the host tissues [14]. For example, quantitative analysis of fungal biomass was used in the evaluation of the relationship between fungal colonization and disease development [18], in the comparison of fungal colonization of different plant organs [19] and in studies of the infection mode of a fungal pathogen [12].

PCR is the method of choice for species-specific detection of fungi in plant material [20]. Amplification products are traditionally separated by electrophoresis, which does not allow for the quantitative analysis of template DNA. Quantification of DNA template can be achieved by competitive PCR, which is based on the use of internal standards co-amplified with target DNA in the same tube using the same primer pair. The ratio of the intensities of the two products serves as a measure of the amount of the template. Nicholson’s and Jenkinson's groups developed competitive PCR assays for several *Fusarium* species [18, 21] and used them successfully in a series of projects, among others to address the effects of fungicides on DON production [22–23]. However, competitive PCR never became a routine method because it is labor-intensive and has a limited throughput.

Real-time PCR allows species-specific quantification within a range of several orders of magnitude and is capable of processing several hundred samples per instrument per day. Reliable species-specific PCR primers are known for all FHB-relevant species [14, 18, 24–25].

Most currently used qPCR methods for *Fusarium* spp. are based on TaqMan technology and commercial DNA extraction kits. Because the distribution of *Fusarium* spp. DNA in kernels is highly inhomogeneous, the small amount of plant material traditionally used to extract DNA for PCR assays (usually 100 mg) causes a large sampling error. Here we describe an upscaled, cost-effective DNA extraction method and an inexpensive real-time PCR assay for *F. culmorum* and *F. graminearum* based on SYBR Green I fluorescence detection.

## 2. Materials and Methods

### 2.1. Fungal cultures

The fungal strains used are listed in Table 1, the sources and origins of the isolates are specified in Brandfass and Karlovsky [26]. Cultures were maintained on SNA medium (0.5 g/L MgSO_4_ × 7 H_2_O, 1 g/L KNO_3_, 0.2 g/L sucrose, 0.2 g/L glucose, 0.5 g/L KCl, 1 g/L KH_2_PO_4_ and 15 g/L agar). *Fusarium* cultures for DNA extraction were grown for 10 days in 100 mL potato dextrose broth (PDB, 24 g/L) at 24 °C, mycelium was harvested by filtration and freeze-dried.

### 2.2. DNA isolation from pure fungal cultures grown in liquid media

We used a variant of the CTAB method [27], modified in our laboratory as follows. The lyophilized mycelium (200 mg) was pulverized in a mortar with a small amount of silica sand. The ground mycelium was transferred into a 50-mL centrifugation tube containing TES buffer (10 mL, 100 mM Tris, 20 mM EDTA, 1% (w/v) SDS, pH set to 8.0 with HCL) and proteinase K (4 mg). The lysis mixture was incubated at 45°C for 45 min and the content of the tubes was mixed by turning the tubes every 10 min. Subsequently, 5 M NaCl (3.9 mL) was added and the sample was mixed before adding 10% (w/v) cetyltrimethylammonium bromide (1.4 mL, CTAB, Merck, Darmstadt, Germany). The samples were incubated for 10 min at 65°C, cooled in an ice/water bath, and then chloroform-isoamyl alcohol (24:1, 10 mL) was added. After mixing the emulsion thoroughly, the tubes were kept over night in an ice/water-bath. The upper phase (including a small part of the lower phase) was transferred into another centrifugation tube by pipetting and spun for 20 min at 4,000 g (5°C). The aqueous phase was transferred to a new tube containing isopropanol (10 mL) at room temperature, mixed thoroughly and centrifuged for 10 min at 4,000 g and room temperature. The supernatant was decanted and the pellet was rinsed with 70% (v/v) ethanol, dried and dissolved in TE (4.5 mL, 10 mM Tris, 1 mM EDTA, pH set to 8.0 with HCl). In spite of the large volume of buffer used to dissolve the DNA, the process took 6 h or longer in some extractions. Dissolving the pellet can be speeded up by heating the tubes to 40°C and then mechanically destroying the pellet. Undissolved material was removed by centrifugation. DNA was concentrated by ethanol precipitation (1/10 vol. of 5 M ammonium acetate and 2.5 vol. of 96% (v/v) ethanol) and dissolved in TE (0.5 mL).

Quality and quantity of DNA were assessed by electrophoresis in 0.8% (w/v) agarose gels (Cambrex, Rockland, ME, USA) prepared in TAE buffer (40 mM Tris, 1 mM EDTA, pH set to 8.5 with acetic acid). The electrophoresis was carried out at 4 V/cm for 60 min. Double-stranded DNA was stained with ethidium (ethidium bromide, 2 mg/L). Gels were documented with the help of a digital imaging system (Vilber Lourmat, Marne la Vallee, France). Densitometry values were compared with those of lambda phage DNA (methylated, from *Escherichia coli* host strain W3110). The densitometry was performed using Multi Analyst-Software (BioRad, Hercules, CA, USA).

### 2.3. Preparation of plant samples

Wheat grain samples (88–92% dry matter) were separated from the chaff by airflow and sieve cleaning with a stationary threshing machine, paying attention to completely remove rachides and glumes. Five hundred gram-portions of grain were ground in a cross hammer mill (Cross Beater Mill SK 1, bottom sieve 2 mm, Retsch, Haan, Germany), the flour was mixed and a sub-sample (40 g) was frozen at −20 °C.

Maize debris meal was prepared by drying basal stems (segments spanning 20 to 30 cm upwards the onset of the adventitious roots) after the removal of leaves until the relative humidity of the samples dropped below 12%. Then the samples were chaffed and ground with the cross hammer mill, bottom sieve 2 mm, before sub-samples were frozen at −20 °C.

### 2.4. Upscaled DNA extraction from plant material

To extract DNA from ground plant samples, wheat flour (1 g) or maize debris flour (500 mg) were blended in a 50 mL-tube with CTAB-buffer (10 mL, 10 mM Tris, 20 mM EDTA, 0.02 M CTAB, 0.8 M NaCl, 0.03 M N-laurylsarcosine, 0.13 M sorbitol, 1% (w/v) polyvinylpolypyrolidone (Merck, Darmstadt, Germany), pH set to 8.0 with NaOH. Mercaptoethanol (20 μL) and proteinase K (0.2 mg, from a stock solution 20 mg/mL) were added shortly before use. The mixture was treated for 5 sec in an ultrasonic bath (Sonorex RK 100, Bandelin, Berlin, Germany). After an initial incubation period of 10 min at 42 °C and a second incubation for 10 min at 65°C, during which the content of the tubes was mixed every 3 min, chloroform-isoamyl alcohol (8 mL, 24:1) was added. The samples were then thoroughly emulsified, incubated for 10 min on ice and centrifuged for 10 min at 5,000 g at room temperature. A portion of the upper phase (600 μL ) were transferred to a 1.5-mL tube containing a 30% (w/v) PEG (Serva, Heidelberg, Germany) solution (194 μL) and 5 M NaCl (100 μL), mixed, and centrifuged for 15 min at 15,000 g at room temperature. The pellet was washed with 70% (v/v) ethanol, dried and dissolved in TE (200 μL). To ensure that the DNA was dissolved completely, the sediment covered by the TE buffer was incubated over night at 4°C. The quality and concentration of DNA were assessed by agarose electrophoresis as described above. A 1:10-dilution was used in the PCR.

### 2.5. Preparation of standards for quantitative real-time PCR

DNA standards and DNA from unknown samples have to be amplified under identical conditions. Therefore, quantified standard-DNA of *Fusarium* spp. was mixed with DNA extracted from uncontaminated wheat flour to imitate matrix effects. A dilution series from 0.5 pg to 0.5 ng of *Fusarium* spp. DNA with a dilution factor of 10 was produced separately for DNA of *F. culmorum* DSM 62191 and *F. graminearum* CBS 389.62 dissolved in the matrix. For each pathogen two standard curves were set up, one in wheat flour matrix and another for maize debris matrix. In addition to the standard curve, two negative controls were processed in each PCR set, one containing no template (water) and one containing 50 pg of the other *Fusarium* species (e.g., *F. graminearum* for *F. culmorum* analysis) mixed with DNA of a healthy plant. Two replicas of each standard were used in each assay. In Table 2 the setup for standards for *F. culmorum* in wheat flour is shown as an example.

### 2.6. Real-time PCR

The iCycler System (BioRad, Hercules, CA, USA) was used for the amplification and quantification of *Fusarium* spp. DNA in plant samples. Primers Fg16N F (ACAGATGACAAG ATTCAGGCACA) and Fg16N R (TTCTTTGACATCTGTTCAACCCA) were used to amplify a 280 bp fragment specific for *F. graminearum* [18]. Primers OPT18 F (GATGCCAGACCAAGACGAAG) and OPT18 R (GATGCCAGACGCACTAAGAT) served to multiply a 472 bp fragment specific for *F. culmorum* [28]. Both primer pairs were derived from randomly amplified genomic fragments, the function of the target sequences is unknown.

The amplification mix for *F. culmorum-*specific PCR consisted of NH_4_-reaction buffer (16 mM (NH_4_)_2_SO_4_, 67 mM Tris-HCl, 0.01% (v/v) Tween-20, pH 8.8 at 25°C; Bioline, Luckenwalde, Germany), 4 mM MgCl_2_, 0.2 mM of each dATP, dTTP, dCTP and dGTP (Bioline, Luckenwalde, Germany), 0.3 μM of primer OPT18 F and OPT18 R, 0.25 u BIOTaq DNA polymerase (Bioline, Luckenwalde, Germany), 10 nM fluorescein (BioRad, Hercules, CA, USA; to facilitate the collection of well factors), 0.1x SYBR Green I solution (Invitrogen, Karlsruhe, Germany), template DNA (1 μL) and doubly distilled water (ddH_2_O) filled to a total volume of 25 μL. The amplification mix for the *F. graminearum-*specific PCR consisted of 1x SYBR Premix Ex Taq (containing TaKaRa Ex Taq HS, dNTP Mixture, Mg^2+^, and SYBR Green I, Takara Bio, Otsu, Japan), 0.3 μM of primer Fg16N F and Fg16N R, 10 nM fluorescein, template DNA (1 μL) and ddH_2_O filled to 25 μL. The detection of amplification products, based on the fluorescence of SYBR Green I, was performed with filters set at 490±10 nm for excitation and 530±15 nm for emission.

The PCR was performed according to the following cycling protocol. Initial denaturation for 1.5 min at 95°C (the denaturation time is used by the thermocycler to collect data for the calculation of the well correction factors, which is needed to compensate for differences among wells of the microtiter plate) was followed by 35 cycles with 30 s at 94°C, 45 s at 64°C, and 45 s at 72°C. The final elongation was performed for 5 min at 72°C. Fluorescence was determined during the annealing step of each cycle. Following amplification, the melting curves were acquired by heating the samples to 95°C for 1 min, cooling to 55°C for 1 min and then slowly increasing the temperature from 65°C to 95°C at the rate of 0.5°C 10 s^−1^, with continuous measurement of the fluorescence.

Electrophoretic analysis of PCR products during the optimization was performed by mixing 4 μL of reaction products with loading buffer (2 μL, 100 mM EDTA, 50% (v/v) glycerol, 0.025% (w/v) bromphenol-blue) and loading the sample on a 1.7% (w/v) agarose gel prepared in TAE buffer. The electrophoresis was carried out as described above.

### 2.7. Fusarium DNA quantification in plant material

Fluorescence data were exported from iCycler software using the "Reports" function within the "PCR Standard Curve" menu. To convert the DNA quantity in pg determined in a PCR tube to the concentration of fungal DNA in the plant sample (μg DNA per kg plant material), the value obtained by real-time PCR had to be multiplied with a factor of 33.333 and 66.667 for wheat flour and maize debris, respectively. (Different factors for wheat and maize samples were needed because of the difference in the sample size used in the optimized extraction protocol, see section *Upscaled DNA extraction from plant material* above).

### 2.8. Box plot

Box plot in Figure 2 was used to graphically represent statistical parameters for the comparison of the reproducibility of DNA extraction. Boxes indicate the middle 50% of the data with the median (solid line) and the mean (dashed line). The whiskers extend to the most distant data points within the 1.5-fold interquartile distance. Data points outside these intervals are denoted by single dots. The minimum and maximum values are printed below and above the box plot, respectively.

## 3. Results and Discussion

### 3.1. Upscaling and optimization of DNA extraction

Our goal was to optimize DNA extraction for invariable yield and constant PCR efficiency. We started with samples of 100 mg wheat flour in 1 mL CTAB buffer using a protocol developed for agar plaques [26] and observed a large variation among replicas. The same was true for maize stem flour, prepared from residues collected in the field after the harvest or in the spring. Furthermore, coprecipitation of some components of wheat flower (probably starch) with DNA generated large pellets which were difficult to dissolve.

Mulfinger *et al*. [29] reported that sampling technique has a major influence on the analytical results in *Fusarium* contamination studies. According to these authors, sample size is a critical factor in the quantification of fungal biomass in plant samples, as fungal growth is inhomogeneous within a kernel and variable among kernels. To reduce the effect of this variability, we upscaled the DNA extraction procedure as follows. For each sample, 500 g of wheat was ground in a hammer mill and one gram of the resulting flour was used for DNA extraction with 10 mL of CTAB buffer. The homogenization in a reciprocal mill used in the original protocol was replaced with a short treatment in an ultrasonic bath.

We found that smaller and easier to dissolve DNA pellets were formed when the chloroform-isoamyl extraction was performed in ice/water-bath for 10 minutes, probably because of increased amount of protein removed in this step. Furthermore, shortening the PEG precipitation step improved the reproducibility of PCR amplification; therefore the centrifugation was started immediately after the DNA extract was vigorously mixed with PEG/NaCl solution. In order to prevent PCR inhibition by substances co-extracted with DNA [30], DNA dissolved in 200 μL TE was diluted 10-fold with distilled water and one microliter of the diluted solution was used as a template for PCR. Increasing the amount of DNA has not improved the detection limit with DNA prepared according to our CTAB protocol nor with DNA extracted with DNeasy Plant Mini Kit (Qiagen, Hilden, Germany). Presumably, PCR inhibitors extracted from plant material were not completely eliminated by the purification steps incorporated into these protocols.

**Figure 1.**
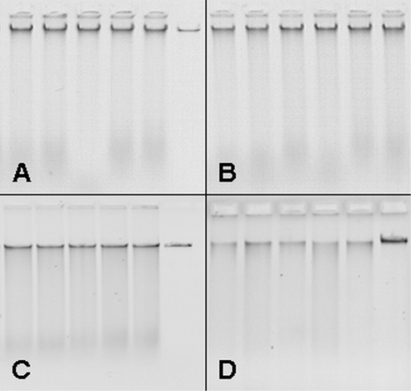
Yield and quality of DNA extracted from wheat flour and maize stem residues. (A) Lane 1–5: healthy wheat kernels (five identical samples), lane 6: 25 ng λ-DNA. (B) Lane 1–6: infected wheat kernels. (C) Lane 1–5: healthy maize stem residues, lane 6: 25 ng λ-DNA. (D) Lane 1–5: infected maize stem residues, lane 6: 25 ng λ-DNA. Four microliters (from a total volume of 200 μL) of DNA extracted from 1 g of wheat flour or 500 mg of ground maize debris according to a modified CTAB protocol (see Material and Methods) were loaded on a 0.8% agarose gel and separated at 4 V/cm for 60 min.

**Figure 2.**
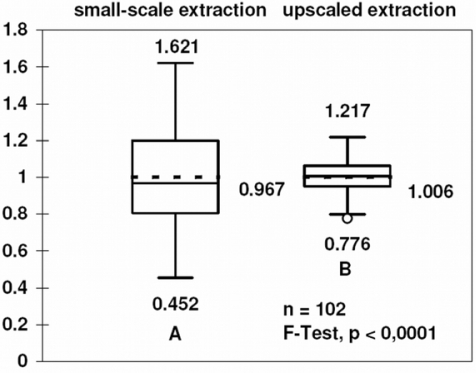
Comparison of the the original and upscaled DNA extraction method. Each of 102 naturally infected wheat kernel samples was extracted twice using a small-scale CTAB method [26] and an upscaled optimized protocol (Materials and Methods). DNA concentration was quantified by densitometry after agarose gel electrophoresis. The y-axis represents the relative yield related to the mean. The box covers the second to third quartile. The mean, maximum value and minimum value are printed. For the definition of the intervals above and below the box see Materials and Methods section.

The modified DNA extraction procedure generated high quality DNA with a low amount of RNA (Figure 1). Even partially degraded DNA from maize debris was obtained in a quality suitable for PCR analysis. The variability of the yield of the original protocol as compared with the upscaled method is shown in Figure 2. The variance of the yield for the two methods differed significantly (F-test). The relative standard deviation was reduced from 0.235 before the optimization to 0.089 after upscaling and optimization.

The DNA extraction procedure is described in detail in the Materials and Methods section. DNA extracted according to other protocols (for example, [31]) or with a commercial kit is likely to be suitable for our PCR assays, too. Upscaling the amount of starting material from a common amount of 50–100 mg to 1 g, however, would increase the costs prohibitively if a commercial DNA extraction kit is used.

### 3.2. Optimization of PCR conditions

Our initial PCR experiments were done under the conditions described in the literature for classical PCR with primers Fg16N and OPT18 [18, 28]. Because these conditions led to poor performance in the real-time mode, we re-optimized primer concentration, polymerase activity, MgCl_2_, and SYBR Green I concentrations and cycling parameters, using native Taq polymerase (BioTaq, Bioline, Luckenwalde, Germany). The performance of the optimized assay for *F. culmorum* was satisfactory, but the sensitivity of the *F. graminearum* assay was not adequate for wheat flour and unspecific products were formed in samples with a low concentration of *F. graminearum* DNA. After comparing different commercial kits and ready-made PCR cocktails available on the market, we found that "SYBR Premix Ex Taq" (Takara Bio, Otsu, Japan) offered the best performance for the *F. graminearum* assay. As the supply of thermostable polymerases constantly changes, the suitability of new brands of polymerase and/or PCR cocktails has to be determined before they are incorporated into the assays.

The specificity of the primers selected for the assay was evaluated in the literature as follows: Fg16N was tested with 21 *F. culmorum* isolates, 24 *F. graminearum* isolates, 20 isolates of other *Fusarium* species and 5 isolates of other fungal species associated with cereals [18]. O’Donnell *et al*. [32] proposed the existence of eleven phylogenetically distinct lineages (regarded as species by some authors) within the *F. graminearum* clade (*F. graminearum* sensu lato). Lineage 7 (syn. *F. grami-nearum* sensu stricto) dominates in wheat and maize in Europe [33–34]. Primer pair Fg16N F/R amplifies part of the sequence amplified with the Fg16 F/R primer set [18]. According to Waalwijk et al. [10], *F. graminearum* lineages 1, 2, 6 and 7 were amplified using the Fg16 F/R primer set, whereas no PCR products were detected for lineages 3, 4 and 5.

*F. culmorum*-specific primers OPT18 were tested in the original publication with 69 *F. culmorum* isolates, 34 *F. graminearum* isolates, 25 isolates of other *Fusarium* species and 27 isolates of other fungal species associated with cereals [28]. All tests confirmed the specificity of OPT18 for *F. culmorum.*

Because PCR conditions used in the specificity tests reported in literature were different from the conditions of our assay, we re-evaluated the specificity of both primer sets. A range of fungal species regularly encountered on cereals was tested (Table 1). All *F. culmorum* and *F. graminearum* Schwabe isolates (12 isolates of each species) tested positively in the corresponding assays, generating PCR products with expected melting temperatures. There was no cross-reaction between *F. culmorum* and *F. graminearum*. All other fungal isolates listed in Table 1 tested negatively in both assays. The amplification of *F. culmorum* and *F. graminearum* was not inhibited by the presence of a large excess of wheat DNA (10 ng wheat DNA in a reaction containing 10 pg of *Fusarium* spp. DNA) nor did pure wheat DNA generate any signal.

### 3.3. Matrix effects and calibration

As in any indirect analytical method, the quality of the standard curve is crucial for quantitative real-time PCR. Specific plant DNA sequences co-amplified with fungal DNA fragments [35] could not be used as an internal standard because plant DNA in maize debris was degraded by saprophytes to a variable degree (data not shown). We suggest that mixing defined amounts of fungal mycelium with uncontaminated plant material is the best way of preparing standards for fungal biomass determination, but the amounts of *Fusarium* spp. in our samples were very low, rendering the preparation of adequate mycelium/plant mixtures difficult. We therefore used pure fungal DNA dissolved in the appropriate matrix for the construction of standard curves. In order to avoid the bias inherent to the calculation of DNA concentration from the optical density of DNA solution at 260 nm [36–37], the concentration of *F. graminearum* and *F. culmorum* DNA extracted from pure fungal cultures was determined by densitometry after agarose electrophoresis, using DNA of bacteriophage Lambda as a standard.

DNA extracts of healthy wheat kernels and uninfected maize stalks from the greenhouse were used as a matrix in which defined amounts of pure fungal DNA were dissolved to serve as standards (see *Preparation of standards for quantitative real-time PCR* part of Material and Methods section for details). For wheat flour, the calibration curve represented a range of 167 ng to 16.7 μg *Fusarium* spp. DNA per gram material. The standard curve for maize stem flour ranged from 333 ng to 33.3 μg *Fusarium* spp. DNA per gram material, because only 500 mg of ground maize stem material were used (see *Upscaled DNA extraction from plant material* part of Material and Methods section for details). The limit of quantification for both assays was estimated to be 0.5 pg genomic DNA, corresponding to about ten *F. graminearum* genomes. This is similar to the values reported for TaqMan assays: Waalwijk *et al*. [15] reported 0.9 pg, Yli-Mattila *et al*. [17] reported 0.1 pg and Reischer *et al*. [25] specified the sensitivity of their assay as 5 genomes. The upper limits of the detection fell outside the standard curve, but the concentration of DNA in field sample extracts never exceeded the the highest DNA concentration used in the standard curve. For comparison, a maize cob fully colonized by *F. graminearum* after artificial inoculation contained 143 μg *F. graminearum* DNA per gram.

**Figure 3.**
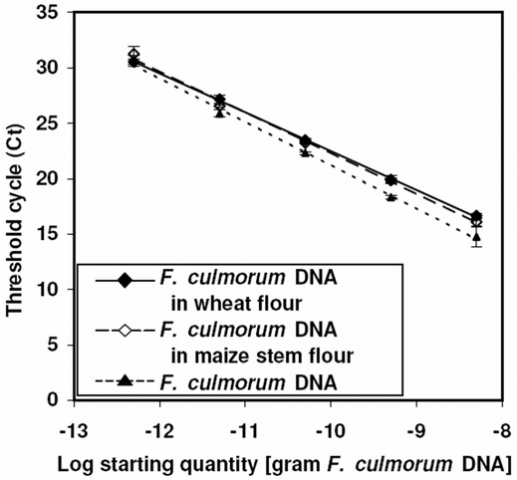
Standard curves used for the quantification of *F. culmorum* by real-time PCR in different matrices. PCR was performed with primer pairs specific for *F. culmorum* and SYBR Green I fluorescence detection as described in Materials and Methods. Standard curves consisted of 10-fold serial dilutions from 0.5 pg to 5 ng *F. culmorum* DNA in matrices as specified in the inlet. Threshold cycles (Ct) means and standard deviations of three replicas were plotted against the amount of *F. culmorum* DNA.

Typical calibration lines for *F. culmorum* DNA in maize and wheat matrices are shown in Figure 3, along with standards for pure *F. culmorum* DNA. R*^2^* (coefficient of determination) for the three calibrations were: 0.996 for maize stem debris, 0.999 for wheat flour and 0.996 for water. The significant effect of the matrix on PCR efficiency demonstrated that the use of matrix extracts in calibration standards was necessary. The effects of both matrices were similar; in spite of this, we strongly recommend to prepare calibration standards in the genuine matrix for each kind of samples. A set of standards in adequate matrix should be run with each PCR plate.

### 3.4. Comparison with other methods

Most of the real-time PCR assays for *Fusarium* spp. available so far rely on DNA extraction from small amount of plant material (typically 50–100 mg) and utilize doubly-labeled probes in combination with kit-based DNA extraction. A comparison of the costs of consumables used in different protocols is given in Tables 3 and 4. The major feature of the DNA extraction protocol developed in this work is the use of larger amounts of starting material, which significantly diminished sampling error (Figure 2). As compared with commercial DNA extraction kits, the protocol reduces the costs of DNA extraction from 1 g material to 6% (Table 3). The saving is significant even when the protocol is compared with small-scale DNA extraction kits. The working time was similar for our protocol and DNeasy Plant Kit (Qiagen) except for drying and dissolution of DNA in the final step.

The use of SYBR Green-based detection eliminates the costs of doubly-labeled hybridization probes (e.g., TaqMan), which makes up 10% of the total costs of the PCR. Apart from doubly-labeled hybridization probes, the major cost-affecting components of PCR are DNA polymerase and nucleotide triphosphates. The demand for high-quality enzymes (hot-start modification, high processivity) tends to grow with the complexity of the method. For example, all TaqMan assays for *Fusarium* spp. developed by Waalwijk *et al*. [15], Reischer *et al*. [25] and Yli-Mattila *et al*. [17] used hot start polymerase, while our SYBR Green-based assays used hot-start polymerase for *F. graminearum* and low-priced native Taq polymerase for *F. culmorum*. The costs of TaqMan assays can theoretically be reduced by multiplexing, but this option has rarely been used because competition among templates may suppress the detection of a sparse template in the presence of an abundant template. Yli-Mattila *et al*. [17] developed a multiplex real-time PCR assay for *Fusarium* spp., but they have not systematically investigated the effect of templates presented in large amounts on the detection of templates presented in low amounts. The fact that more cycles were needed to reach the threshold value in their multiplex PCR assay as compared to PCR with only one primer pair and a probe indicates that competition among templates indeed occurred. As long as TaqMan assays are performed in a simplex form, they are more costly than SYBR Green-based assays.

As the specificity of SYBR Green assays relies solely on PCR primers, it is mandatory to check melting curves of PCR products after real-time detection. Typically primer dimers and other unspecific products only occur in significant amounts in samples with very low amounts of the template. This observation can likely be explained by the competition of low-efficiency amplification of unspecific products with high-efficiency amplification of the template, for which PCR conditions have been optimized. Therefore, examination of melting curves after SYBR Green assays is particularly important for samples with high threshold cycle values (lower end of the calibration curve).

## 4. Conclusions

Sampling error in DNA extraction from wheat grain flour and maize stalks for real-time PCR was reduced considerably by upscaling the protocol from 50–100 mg to 1 g plant material. Because the costs of commercial DNA extraction kits for this sample size are prohibitive, the use of a cetyl-trimethylammonium-based (CTAB) protocol is recommended. Species-specific real-time PCR assays for *Fusarium graminearum* and *Fusarium culmorum* with the detection of SYBR Green fluorescence proved to be a suitable alternative to the use of TaqMan and other kinds of doubly-labeled hybridization probes. Matrix effects have to be compensated by preparing calibration standards in plant extracts. The combination of the upscaled CTAB-based DNA extraction protocol with SYBR Green-based real-time PCR offers a cost-effective method for the estimation of *F. graminearum* and *F. culmorum* biomass in plant material.

## Figures and Tables

**Table 1.** Fungal strains.

Species	Number of Isolates
*Alternaria alternata* (Fries) Keissler	1
*Cladosporium herbarum* (Pers.) Link Ex SF Gray	2
*Drechslera sorokiniana* (Sacc.) Subram. & Jain	1
*Fusarium acuminatum* Ellis & Everh.	5
*Fusarium avenaceum* (Fr.) Sacc.	2
*Fusarium compactum* Wollenw.	1
*Fusarium crookwellense* Burgess, Nelson & Toussoun	3
*Fusarium culmorum* WG Smith	12
*Fusarium equiseti* (Corda) Sacc.	5
*Fusarium graminearum* Schwabe	12
*Fusarium oxysporum* (Schlech.) Snyder & Hansen	1
*Fusarium poae* (Peck) Wollenw.	2
*Gibberella fujikuroi* (Saw.) Wr.	5
*Microdochium nivale* (Fries) Samuels & Hallett var*. majus*	1
*Microdochium nivale* (Fries) Samuels & Hallett var. *nivale*	3
*Pseudocercosporella herpotrichoides* (Fron) Deighton	3
*Rhizoctonia cerealis* Van der Hoeven	3
*Stagonospora nodorum* (Berk.) Castellani & Germano	1

**Table 2.** Standards for the quantification of *F. culmorum* DNA in wheat flour.

Sample type	Sample No.	*Fusarium* spp. DNA
Negative controls	1	-
	2	50 pg *F. graminearum* DNA in wheat flour matrix diluted 1:10
Standards	3–4	0.5 pg *F. culmorum* DNA in wheat flour matrix diluted 1:10
	5–6	5 pg *F. culmorum* DNA in wheat flour matrix diluted 1:10
	7–8	50 pg *F. culmorum* DNA in wheat flour matrix diluted 1:10
	9–10	0.5 ng *F. culmorum* DNA in wheat flour matrix diluted 1:10

**Table 3.** Comparison of costs of consumables for DNA extraction. Average price for spin column-based products of two leading suppliers was used.

DNA extraction method	Sample size	Costs per sample
Commercial kit	50–100 mg	1.80–2.60 €
	1 g	8.00–10.00 €
CTAB based protocol described in this work	1 g	0.50 €

**Table 4.** Comparison of the costs of PCR components per sample.

	Taqman probes	SYBR Green-based detection
TaqMan probe	0.08–0.12 €	-
SYBR Green	-	0.001 €
DNA polymerase, dNTPs, primers, PCR tubes and filter tips	0.60–2.20€	
